# Profiling Vulnerability in Youth and Predicting Educational Attainment in Young Adulthood

**DOI:** 10.1002/jad.70042

**Published:** 2025-08-18

**Authors:** Heidi M. Renner, Bosco Rowland, Delyse Hutchinson, John W. Toumbourou

**Affiliations:** ^1^ School of Psychology, Centre for Social and Early Emotional Development (SEED), Faculty of Health Deakin University Geelong Victoria Australia; ^2^ Murdoch Children's Research Institute Melbourne Children's LifeCourse Initiative Parkville Victoria Australia; ^3^ Eastern Health Clinical School & Monash Addiction Research Centre Monash University Richmond Victoria Australia; ^4^ Murdoch Children's Research Institute Centre for Adolescent Health Parkville Victoria Australia; ^5^ Department of Paediatrics, Royal Children's Hospital University of Melbourne Parkville Victoria Australia; ^6^ National Drug and Alcohol Research Centre, Faculty of Medicine University of New South Wales Sydney New South Wales Australia

**Keywords:** disadvantage, early school leaving, equity, social determinants, vulnerability

## Abstract

**Introduction:**

Educational attainment is associated with higher rates of employment, income, and standard of living; yet leaving secondary school before completion of the final year remains common, particularly for youth experiencing disadvantage. This study aimed to identify key indicators of vulnerability, derived from a proposed framework of child disadvantage, that predicted early school leaving in a state‐representative sample of Australian youth.

**Methods:**

Data comprised 2884 participants (51.7% female; 48.3% male) across three age cohorts from the Australian arm of the longitudinal cohort study, the International Youth Development Study (IYDS). The relationship between level of vulnerability in adolescence (11–15 years old in Wave 1; 2002) and subsequent early school leaving (19–23 years old in Wave 7; 2010) was examined, controlling for individual, family, school, and community covariates.

**Results:**

Latent class analyses identified four vulnerability groups (‘low,’ ‘normative,’ ‘welfare,’ and ‘high’), differentiated by sociodemographic factors (low), receipt of welfare support (welfare), and family and community risk factors (high). Multivariate regression analyses indicated greater vulnerability in adolescence (11–15 years old) predicted an increased odds of subsequent early school leaving, with the highest vulnerability group 40% more likely to leave school before completing Year 12, relative to the lowest vulnerability group (*OR* = 1.40; 95% CI [1.27, 1.53], *p* < 0.001).

**Conclusions:**

Sociodemographic, geographical, and risk indicators, selected using a multidimensional framework of child disadvantage, predicted increased vulnerability for early school leaving. Prevention and intervention initiatives should select comprehensive multidimensional indicators to prioritise vulnerable youth with the aim of improving educational equity.

## 1 Research Background

Educational disparities can have substantive impacts on work opportunities and career trajectories for young people (Almquist 2016; Lamb 2011). Youth who leave school early, before completion of secondary school, are less likely to progress to further education, are at greater risk of long‐term unemployment, poorer physical and mental health (Robinson and Meredith 2013) and are more likely to engage in antisocial behaviour (Hemphill et al. 2010). While some young people who leave school early engage in further training and follow vocational pathways, and may benefit from leaving the school environment, most young people do not engage in other forms of education or training (Nether in Education, Employment of Training; NEET) and find themselves on poorer employment trajectories (Lamb et al. 2020; The Smith Family 2024). In Australia in 2022, nearly a quarter of secondary students (24%) left school before the completion of the final year of secondary education (Australian Curriculum Assessment and Reporting Authority 2025). For these youth, understanding the factors associated with early school leaving is therefore important to inform approaches to reduce educational inequities and improve developmental outcomes.

Some factors, such as social and economic circumstances, are not distributed equally across society and can impact the life course development of an individual. These circumstances, often referred to in the literature as the *social determinants of health*, are conditions a person is born into and are often difficult to modify (Commission on Social Determinants of Health 2008; Marmot et al. 2008). Disparity between those who are wealthy and poor, has been linked to a range of structural determinants of disadvantage, typically present from birth, such as access to healthcare, work opportunities, and suitable living conditions. In Australia, educational inequity is demonstrated through secondary school completion rates that vary according to socioeconomic status (70% low SES to 83% high SES) and geographical location (64% regional/remote vs 79% major city; Australian Curriculum Assessment and Reporting Authority 2025). This is also reflected in the completion rates across Australia, where states and territories with more remote communities have lower rates of school completion (e.g., 56% in the Northern Territory compared to 84% in Victoria). Disparity in access to education is also recognised as an important social determinant globally, contributing to poorer health outcomes and education pathways for youth experiencing disadvantage (Allee‐Herndon and Roberts 2019; Marmot et al. 2008; Wilson and Tanner‐Smith 2013).

Inequitable pathways can arise due to environmental factors and historical influences on an individual. Psychosocial factors associated with school completion and early school leaving are evident across multiple domains of a child's life and can be grouped across key areas (Gubbels et al. 2019): (a) physical and mental problems (e.g., poor mental health) (Almquist 2016; Bowman et al. 2017); (b) antisocial behaviours (Almquist 2016); (c) substance use (Silins et al. 2015; Townsend et al. 2007); (d) peer group characteristics (e.g., prosocial or antisocial peers) (Goza and Ryabov 2009; Rendón 2014); (e) parenting problems (Barker et al. 2017; Chau et al. 2022); (f) family structure (Roos and Wall‐Wieler 2017); (g) problems at or with school (e.g., prior academic achievement or failure, suspension and expulsions) (Suh et al. 2007); (h) characteristics of the school (e.g., low level of school connectedness) (Bond et al. 2007), (i) sociodemographic factors (e.g., parental education and income or family socioeconomic status (SES) (Kallio et al. 2016; Suh et al. 2007); and (j) cultural factors (e.g., acculturation and stereotype threat) (Kao and Thompson 2003; Weber et al. 2018). Each of these areas represent a complex range of factors that can influence education. For example, at the school level, low school connectedness and school poverty (Fasang et al. 2014; Kitchin and Karlin 2024) and “push” factors such as suspension or expulsion (Doll et al. 2013) can increase early school leaving. Individually, these factors account for some proportion of early school leaving, however understanding a child's accumulation of risk across multiple environmental domains (i.e., individual, peer, family, school, and community), and the context in which these risks occur (political and economic, school and system, and student context), may enable a more comprehensive understanding of the modifiable influences on educational attainment and early school leaving (Bronfenbrenner and Morris 2006; Lamb 2011; Gubbels et al. 2019; Schoon and Melis 2019). Through understanding the multifaceted nature of disadvantage and ensuring measurement captures this complexity, interventions can be designed and tested that address the social, economic, and systemic drivers that underpin inequality (Wake et al. 2022).

A recent multidimensional framework of child disadvantage that modelled social determinants (i.e., sociodemographic, geographical environments, health conditions, and risk factors) across ecological levels (individual, family, and community) to operationalise and measure child disadvantage, offers a promising approach to understand disadvantage in young people in Australia (Goldfeld et al. 2018). This framework, developed in a nationally representative sample of Australian children aged 8–9 years, suggests that child disadvantage is best conceptualised and measured through a social determinants lens. The framework seeks to inform policy through first understanding the pattern of the drivers of child disadvantage to then identify modifiable points for intervention to improve health and educational equity (e.g., academic achievement). This aligns with international evidence that proposes child disadvantage exists on a continuum, with those affected by higher levels of disadvantage experiencing poorer health and developmental outcomes, including poorer academic outcomes (Marmot et al. 2008). Identifying whether this framework can be applied to other development age groups and identifying ways to disrupt poorer trajectories and improve educational outcomes is therefore an important research area to subsequently inform educational interventions.

Current international interventions to improve school retention show promise for prioritising youth exposed to disadvantage (Hahn et al. 2015; Rodríguez and Conchas 2009; Wilson et al. 2011; Wilson and Tanner‐Smith 2013), however much of the literature measures disadvantage using single factors. Identifying a group of factors, through a multidimensional lens, that capture the complexity of disadvantage across contexts and environments may better support vulnerable youth. Applying the proposed framework of child disadvantage (Goldfeld et al. 2018) at different ages and with different outcomes may enable thorough measurement, embedded in theory, to encompass the complexity of vulnerability for early school leaving. It may also extend theory in this area through replication of this proposed framework. The Communities That Care (CTC) youth survey uses a multidimensional lens to measure risk factors for school failure (Feinberg et al. 2010), and other health and social problems (Toumbourou et al. 2019) and may align with the proposed framework (Goldfeld et al. 2018) for measuring vulnerability. This may help governments and policy makers to identify and prioritise vulnerable groups and to guide preventative interventions.

## 2 Research Purpose

The overarching purpose of the present study was to use the Australian adaptation of the CTC youth survey to identify the profile of indicators, in a state‐representative sample of Australian youth, that predict vulnerability for early school leaving (i.e., leaving school before the completion of secondary school). As such, the aims were threefold: (a) to apply the Goldfeld et al. (2018) framework of child disadvantage to guide the operationalisation of indicator selection for vulnerability, and to replicate and extend prior research in a different sample of Australian youth (11–15 years old, living in Victoria), with a different academic outcome (early school leaving); (b) to identify classes of youth who vary in vulnerability via latent class modelling; and, (c) to examine the association of varying levels of vulnerability with early school leaving, while adjusting for covariates. Based on prior literature, it was hypothesised that there would be four classes of vulnerability (low, moderately low, moderately high, and high) and that early school leaving would increase as a function of increasing vulnerability level (Almquist 2016; Lanza et al. 2010; Schoon and Melis 2019).

## 3 Methodology

### 3.1 Participants

This study examined data from the International Youth Development Study (IYDS), a prospective longitudinal study that commenced in 2002 in Victoria, Australia, and Washington State, in the United States (McMorris et al. 2007). The IYDS was designed to investigate adolescent development using risk and protective factors to examine substance use and delinquent behaviour. Ethics approval in Australia was originally obtained from the Royal Children's Hospital Ethics in Human Research Committee; the IYDS design and sampling methodology is comprehensively detailed elsewhere (McMorris et al. 2007).

The IYDS in Victoria, Australia involved 10 waves of data collection, from 2002 through to the most recent wave in 2019. A two‐stage cluster sampling approach was used in Wave 1 (at the school and class level) to obtain a representative sample of students from three different year levels (grades 5, 7, and 9). Students were recruited through information packs (which included a plain language statement and consent form) distributed by teachers to students in the selected classes to take home to their parents (McMorris et al. 2007). Of the 3926 eligible students from 152 schools invited to participate in the study, 2884 (73.5%) agreed to participate in the first wave of data collection. In relation to those who refused, parents of the youngest cohort were more likely to refuse participation (26%) than the older cohorts (around 18%–19%). Informed, written, active (opt‐in) consent was obtained from all parents before the first wave of data collection. Individual participants also provided consent before participation at each wave of the study. Wave 1 data was collected through a short telephone interview with participants' parents and via youth self‐report surveys (CTC Australian adaptation) delivered at school (Arthur et al. 2002). The Wave 7 self‐report survey was completed by participants via paper or online surveys, or via telephone interview with researchers.

The current study focused on the Victorian sample of 2,884 young people in three age cohorts across two waves (Wave 1 at baseline and Wave 7 at young adulthood), as outlined in Table 1. The average age at Wave 1 (2002) was 13 years (ranging from 11 to 15 years) and at Wave 7 (2010) was 21 years (ranging from 19 to 23 years). The retention rate between Wave 1 and Wave 7 was 84%. The IYDS sample was a good representation of the Victorian school‐age population, with slightly higher levels of low‐income assistance (welfare; McMorris et al. 2007). See Table 1 for a summary of the sample including key demographic information.

**Table 1:** International Youth Development Study sample size, distribution, retention rates, and Wave 1 demographics for Victorian cohort.

Wave	Cohort											
	Grade 5 Primary school			Year 7 Early adolescence			Year 9 Mid‐adolescence			Total		
	*n*	*M*	SD	*n*	*M*	SD	*n*	*M*	SD	*N*	*M*	SD
Wave 1 (2002)	927	11.0	0.4	984	12.9	0.4	973	15.0	0.4	2884	13.0	1.6
Wave 7 (2010)	809	19.0	0.4	826	21.1	0.5	788	23.0	0.5	2423	21.0	1.7
Missing^a^	118			158			185			461		
Retention rate^b^	87%			84%			81%			84%		

Demographics @ Wave 1	*n*	%		*n*	%		*n*	%		*n*	%	
Gender	927			984			973			2884		
Female	482	52		500	51		508	52		1490	52	
Male	445	48		484	49		465	48		1394	48	
Country of birth	878			948			923			2749		
Australia	831	95		889	94		855	93		2575	94	
Country other than Australia	47	5		59	6		68	7		174	6	
Aboriginal or Torres Strait Islander	12	1		11	1		7	1		30	1	
Parental income^c^	696			780			706			2182		
Less than $30,000	165	24		171	22		151	21		487	22	
Between $31,000 and $60,000	220	32		246	32		206	29		672	31	
Between $61,000 and $100,000	231	33		262	34		225	32		718	33	
Over $100,000	80	11		101	13		124	18		305	14	

*Note:* The mean (*M*) and standard deviation (SD) are presented for the age of each cohort and for waves 1 and 7.

Abbreviations: n, sample size.

^a^ Missing represents participants lost to follow‐up at Wave 7.

^b^ Retention rate represents the proportion of participants retained in the sample between Wave 1 and 7.

^c^ Parental income is in Australian currency (AUD).

## 4 Measures

### 4.1 Vulnerability Indicators

Vulnerability indicators were selected according to the following criteria: (a) aligned with indicators in the Goldfeld et al. (2018) framework of child disadvantage (see Appendix A), and (b) measured in all three age cohorts in Wave 1 of the IYDS. Of existing IYDS indicators, ten fulfilled the above criteria across the social determinants domains of sociodemographic, geographical environment, and risk factors; six were parent‐report and four were youth‐report indicators (see Table 2). Indicators were a combination of dichotomous and continuous measures, and risk factors were validated scales (Hemphill et al. 2011). The IYDS did not include indicators in the health conditions domain thus this domain was not represented in this study. A further three indicators were created to align with the Goldfeld et al. (2018) framework: (a) biological parents of child are partners, (b) community socioeconomic status (SES), and (c) lives in a regional area. The “biological parents of child are partners” indicator was created from parent‐report measures to capture family structure (contribution of single parent households and divorce). The “community SES” and “lives in a regional area” indicators were developed based on the postal codes reported at the time of initial recruitment (parent‐reported) and were created to demonstrate socioeconomic status and geographical location. The “community SES” indicator was created using the Australian Bureau of Statistics (ABS) Index of Relative Socioeconomic Advantage/Disadvantage (IRSEAD), a subcategory of the Socioeconomic Indexes for Areas (SEIFA) (Adhikari 2006). The “lives in a regional area” indicator was created using the ABS definition for *remoteness* (major cities, inner regional, outer regional, remote, very remote, and migratory), developed around the time of data collection (Trewin 2001) and converted to a binary indicator (no/yes). See Appendix B for a full list of indicators and example items.

**Table 2:** Descriptive statistics for vulnerability and school completion indicators.

Indicator	Total		
	Value/Range	*N*	%
Sociodemographic domain			
Annual pretax household income^a^ (*M* = 8.4, SD = 3.9)		2182	
Less than $10,000	1	34	1.2
$10,001–$15,000	2	95	3.3
$15,001–$20,000	3	102	3.5
$20,001–$25,000	4	112	3.9
$25,001–$30,000	5	144	5.0
$30,001–$40,000	6	227	7.9
$40,001–$50,000	7	192	6.7
$50,001–$60,000	8	253	8.8
$60,001–$70,000	9	225	7.8
$70,001–$80,000	10	252	8.7
$80,001–$90,000	11	151	5.2
$90,001–$100,000	12	90	3.1
$100,001–$110,000	13	95	3.3
$110,001–$120,000	14	43	1.5
$120,001–$130,000	15	33	1.1
$130,001–$140,000	16	22	0.8
$140,001–$150,000	17	25	0.9
$150,001–$200,000	18	51	1.8
$200,001 and over	19	36	1.3
Missing		702	24.3
Family speaks language other than English^a^ (*M* = 0.1, SD = 0.4)		2745	
No	0	2360	81.8
Yes	1	385	13.4
Missing		139	4.8
Main caregiver completed secondary school^a^ (*M* = 0.6, SD = 0.5)		2734	
No	0	1091	37.8
Yes	1	1643	57.0
Missing		150	5.2
Main caregiver in paid work^a^ (*M* = 0.8, SD = 0.4)		2741	
No	0	695	24.1
Yes	1	2046	70.9
Missing		143	5.0
Main caregiver/partner recipient of welfare^a^ (*M* = 0.3, SD = 0.5)		2741	
No	0	1904	66.0
Yes	1	837	29.0
Missing		143	5.0
Biological parents of child are partners^a^ (*M* = 0.9, SD = 0.3)		2195	
No	0	207	7.2
Yes	1	1988	68.9
Missing		689	23.9
Number of people in household^a^ (*M* = 4.5, SD = 1.2)		2745	
1	1	5	0.2
2	2	109	3.8
3	3	348	12.1
4	4	1037	36.0
5	5	804	27.9
6	6	325	11.3
7	7	83	2.9
8	8	24	0.8
9	9	5	0.2
10	10	2	0.1
11	11	1	< 0.1
12	12	2	0.1
Missing		139	4.8
Geographical environment domain			
Community disorganisation^b^ (*M* = 1.5, SD = 0.5) (*α* = 0.75)^c^	1–4	2820	
Missing		64	2.2
Community SES^d^ (*M* = 996.5, SD = 74.3)	821.6–1213.3	2872	
Missing		12	0.4
Lives in regional area^d^ (*M* = 0.4, SD = 0.5)		2879	
No	0	1755	60.9
Yes	1	1124	39.0
Missing		5	0.2
Risk factors domain			
Family history of antisocial behaviour^b^ (*M* = 1.8, SD = 0.8) (*α* = 0.77 & 0.79)^c^	1–5	2794	
Missing		90	3.1
Family conflict^b^ (*M* = 2.2, SD = 0.8) (*α* = 0.79)^c^	1–4	2836	
Missing		48	1.7
Number of times changed homes since kindergarten^b^ (*M* = 2.1, SD = 1.1)		2866	
Never	1	1068	37.0
1 or 2 times	2	980	34.0
3 or 4 times	3	511	17.7
5 or 6 times	4	197	6.8
7 or more times	5	110	3.8
Missing		18	0.6
Educational outcome			
Highest year of secondary school completed^b^ (*M* = 1.4, SD = 0.8)		2404	
Year 12 or equivalent	1	1884	65.3
Year 11 or equivalent	2	261	9.1
Year 10 or equivalent	3	190	6.6
Year 9 or equivalent	4	57	2.0
Year 8 or below	5	12	0.4
Missing		482	16.6

*Note:* Means (*M*) and standard deviations (SD) are not standardised.

^a^ Parent‐reported indicator.

^b^ Student/youth‐reported indicator.

^c^ Cronbach's alpha (α) is reported for the full sample except for the “family history of antisocial behaviour” scale where primary and secondary are reported separately.

^d^ Geographic indicator coded from home location.

### 4.2 Early School Leaving

In Australia, school education is compulsory until the age of 16 or 17 years, or completion of Year 10, varying by state and territory, with Year 12 the final year of secondary education. Educational measures and schooling structures vary internationally, thus, to ensure the outcome measure was meaningful in Australia, ABS response options for secondary school completion were used; self‐reported highest year level of secondary school completed was measured with five options (Year 12, Year 11, Year 10, Year 9, and Year 8 or below) (Australian Bureau of Statistics ABS 2009). This study focused on investigating typical school completion as compared to nonschool education and training and therefore did not measure other types of vocational education or training.

### 4.3 Covariates

Factors associated with early school leaving were identified in the literature across eight areas: (a) demographics, (b) ethnicity, (c) problems in school, (d) substance use, (e) antisocial behaviour, (f) childhood emotional and behavioural problems, (g) parenting problems, and (h) mental health problems (Goldfeld et al. 2018; Gubbels et al. 2019; Kao and Thompson 2003; Silins et al. 2015; Suh et al. 2007; Townsend et al. 2007; Weber et al. 2018). Significant covariates in these areas were included in the analyses to control for potential confounding effects (see Appendix B for more detail on the covariates).

### 4.4 Analytic Approach

The analysis involved three steps. First, thirteen IYDS vulnerability indicators that aligned with the Goldfeld et al. (2018) framework were identified and a correlation was conducted to establish independence of the variables. Second, five latent class analysis (LCA) models (two‐ to six‐class) were performed in Mplus version 8.4 (Muthén and Muthén 1998–2017) to identify the most parsimonious and meaningful model fit for the data. Mplus uses full information maximum likelihood (FIML) parameter estimation, based on all the observed data for each case, to account for missing data. Akaike information criterion (AIC), Bayesian information criterion (BIC), log likelihood and entropy model fit statistics were assessed to determine the best fitting model.

Third, negative binomial regression analyses were conducted in StataBE version 17.0 (StataCorp 2021), to identify whether latent classes predicted subsequent early school leaving, with and without adjusting for covariates. StataBE uses listwise deletion of observations to manage missing data resulting in an estimation model that only includes observations with complete data. Negative binomial regression was used as early school leaving was over‐dispersed, and the “svyset” command and “svy” prefix were used to control for clustering by school. Covariates were organised into four levels, according to ecological domains, and entered in the model in a stepped approach from proximal to distal: (1) peer‐individual (proximal), (2) family, (3) school, and (4) community (distal). Nonsignificant predictors were removed from the model at each step, before entering the next level of variables. Lastly, sensitivity analyses were performed to test patterns of missing data using a negative binomial regression of missingness.

## 5 Results

### 5.1 Vulnerability Indicator Selection

The vulnerability indicators and the educational outcome (early school leaving) included in the study are outlined in Table 2. For most participants their main caregiver had completed Year 12 (57%), was in paid work (71%), was not receiving welfare (66%), and lived in a major city (61%). In Wave 7, most participants had completed Year 12 (65%). Risk factors (continuous variables) were positively skewed, with most participants reporting low levels of risk.

Associations between vulnerability indicators showed weak to moderate levels of correlation (see Appendix C). As expected, a stronger correlation between family income and welfare was observed (*r* = −0.61; *p* < 0.001), with lower income associated with welfare support. Indicators were therefore suitable for inclusion in the LCA model as independent indicators. Cronbach's alpha for the 13‐item vulnerability measure was calculated in StataBE version 17.0 (StataCorp 2021) using the “alpha” and “std” commands (i.e., the items were standardised; *α* = 0.55).

### 5.2 Latent Class Analysis of Vulnerability

Model fit statistics indicated the four‐ and five‐class models were the best fitting LCA models (see Table 3). However, the four‐class model also meaningfully differentiated participants with higher vulnerability and was therefore the model used for further analyses.

**Table 3:** Distribution of indicators in the four‐latent‐class model of youth vulnerability in Wave 1 (2002; *N* = 2884) and model fit indices for two‐six‐class modelling.

Indicator	Vulnerability latent classes (C)			
	Low (C1)	Normative (C2)	Welfare (C3)	High (C4)
	18% (*n* = 505)	46% (*n* = 1,337)	24% (*n* = 696)	12% (*n* = 346)
Sociodemographic domain				
Annual pretax household income^a^	12.70	9.43	4.51	6.64
Family speaks language other than English^b^	0.16	0.10	0.21	0.13
Main caregiver completed secondary education^b^	0.90	0.60	0.44	0.46
Main caregiver in paid work^b^	0.84	0.85	0.53	0.66
Main caregiver/partner recipient of welfare^b^	0.04	0.01	0.97	0.47
Biological parents of child are partners^b^	0.96	0.92	0.86	0.79
Number of people in household^a^	4.53	4.55	4.35	4.31
Geographical environment domain				
Community disorganisation^a^	1.38	1.36	1.45	1.98
Community SES^a^	1102.33	976.76	968.19	970.44
Lives in regional area^b^	0	0.50	0.43	0.48
Risk factor domain				
Family history of antisocial behaviour^a^	1.52	1.54	1.61	3.17
Family conflict^a^	2.16	2.05	2.12	2.87
Number of times changed homes since kindergarten^a^	1.96	1.89	2.24	2.46
Attrition by vulnerability class (at wave 7; *n* = 2,404)	10.1%	15.9%	18.4%	25.7%

Number of latent classes	Model fit indices			
	LLV	AIC	BIC	Entropy
2	−47767.21	95602.41	95805.29	0.72
3	−47261.92	94619.84	94906.25	0.79
4	−46803.66	93731.32	94101.27	0.82
5	−46648.21	93448.41	93901.90	0.82
6	−46492.11	93164.22	93701.24	0.81

*Note:* Percentages (%) represent proportions of the wave 1 sample (*N*).

Abbreviations: AIC, Akaike information criterion; BIC, Bayesian information criterion; LLV, Log likelihood value.

^a^ Unstandardised means.

^b^ Probabilities.

The four classes were conceptualised to reflect the following groups: (1) low vulnerability, (2) normative vulnerability, (3) welfare vulnerability, and (4) high vulnerability (Table 3). Class 1, “low vulnerability” (18% of sample), was classified by indicators in the sociodemographic and geographical domains. Individuals in this class had higher levels of sociodemographic advantage; generally, they lived in urban areas with higher SES, and had higher levels of parental income, education, and employment. Class 2, “normative vulnerability,” the largest class (46% of sample), was conceptualised as “normal” or “average” (as per Almquist 2016) with moderate parental income (*M* = 9.43, around $70,000 annually), education (60% completed secondary), and risk factors, as compared to the “high” and “welfare” vulnerability classes. This class was not classified by a specific domain (sociodemographic, geographical, or risk), instead it was defined by moderate levels of the indicators, spread across the three domains. Class 3, “welfare vulnerability,” the second largest class (24% of sample), was also classified by indicators in the sociodemographic domain. Individuals in this class reported the lowest levels of sociodemographic advantage (i.e., they reported the highest levels of sociodemographic disadvantage); generally, they had low levels of household income (*M* = 4.51, around $25,000 annually), parental education (44% completed secondary) and employment (53% employed), and the highest level of welfare support (97% received welfare). Lastly, Class 4, “high vulnerability” (12% of sample), was classified by indicators in the risk factor domain. This class represented individuals with high levels of familial risk factors (family antisocial behaviour and conflict) and community disorganisation (classified as a risk factor in the IYDS), in addition to sociodemographic disadvantage, that is, low parental education (46% completed secondary school) and relatively high welfare involvement (47% received welfare).

### 5.3 Vulnerability Class Prediction of Early School Leaving

Table 4 shows the results of negative binomial regression analyses predicting early school leaving. The risk of early school leaving increased as level of vulnerability increased, with participants in the high vulnerability class (C4) significantly more likely to leave school before completion of Year 12, than those in the low vulnerability class (C1). Participants in the normative (C2) and welfare (C3) classes also had a higher risk of early school leaving compared to those in the low vulnerability class.

**Table 4:** Multivariate negative binomial regression models predicting early school leaving (Wave 7) from vulnerability class (Wave 1), controlling for significant covariates (*N* = 2275).

Variable	*IRR*	95% CI	
		Lower	Upper
Unadjusted model			
Vulnerability class			
C1 ‐ low	Referent		
C2 ‐ normative	1.24***	1.17	1.31
C3 ‐ welfare	1.39***	1.30	1.48
C4 ‐ high	1.63***	1.50	1.77
Adjusted model			
Vulnerability class			
C1 ‐ low	Referent		
C2 ‐ normative	1.18***	1.13	1.25
C3 ‐ welfare	1.31***	1.24	1.40
C4 ‐ high	1.40***	1.27	1.53
Covariates			
Individual domain			
Cohort – middle (referent = youngest)	0.98	0.92	1.05
Cohort – oldest (referent = youngest)	0.84***	0.79	0.89
Student born in Australia	1.14**	1.05	1.23
Interaction with antisocial peers	1.13***	1.07	1.20
Childhood concentration/attention problems	1.09***	1.06	1.12
Family domain			
Parent born in Australia	1.09**	1.03	1.16
School domain			
Suspended from school in past year	1.36***	1.21	1.52

*Note:* Class 1 (low vulnerability) is the comparison group. 95% CI refers to a 95% confidence interval with the lower and upper range reported. This analysis is based on a smaller sample (*N* = 2275) of the Wave 7 sample (*N* = 2404) due to Stata's use of listwise deletion in the regression analysis.

Abbreviation: IRR = Incident Rate Ratio.

** *p* < 0.01

** *p* < 0.001.

Table 5 presents school completion frequencies and percentages for the four classes. Overall, 21.6% of the Wave 7 sample did not complete Year 12 (early school leavers). Relative to the low vulnerability group, characterised by low rates (4.6%) of early school leaving, rates for other classes ranged from 20.0% (‘normative’) to 29.8% (‘welfare’) to 40.9% (‘high’) (based on unadjusted proportions). This relationship persisted after adjusting for the effects of key covariates (see Appendix D for a full list of covariates).

**Table 5:** Frequencies for Wave 7 sample (2010; *N* = 2404), highest year of secondary school completed by predicted class of vulnerability (Unadjusted for confounders).

Highest year of secondary school completed	Vulnerability class (C)								Total	
	Low (C1) 19% (*n* = 454)		Normative (C2) 47% (*n* = 1,125)		Welfare (C3) 24% (*n* = 568)		High (C4) 11% (*n* = 257)			
	*n*	%	*n*	%	*n*	%	*n*	%	*n*	%
Year 12 or equivalent	433	95.4	900	80.0	399	70.2	152	59.1	1,884	78.4
Year 11 or equivalent	12	2.6	118	10.5	86	15.1	45	17.5	261	10.9
Year 10 or equivalent	8	1.8	79	7.0	65	11.4	38	14.8	190	7.9
Year 9 or equivalent	1	0.2	23	2.0	14	2.5	19	7.4	57	2.4
Year 8 or below	0	0	5	0.4	4	0.7	3	1.2	12	0.5
Total (*N* = 2,404)	454	100	1,125	100	568	100	257	100	2,404	100
Missing (*n* = 480)^a^	51	10.1	212	15.9	128	18.4	89	25.7	480	16.4

*Note:* Percentages in the table are calculated from the Wave 7 sample who also responded to the school completion item.

^a^ Missing values are calculated from the difference between Wave 1 and 7 (participants who responded to the education item) for each class, and percentages are calculated from the full sample (*N* = 2884).

Sensitivity analyses indicated that level of vulnerability predicted missingness on early school leaving after adjusting for covariates, with increasing likelihood of missing data as level of vulnerability increased (see Table 6). Participants in the ‘high’ vulnerability category had the highest likelihood to be missing from Wave 7 (or missing a response to the school completion item), as compared to the “low” vulnerability category (*IRR* = 0.90, 95% CI: [0.84, 0.96], *p* = 0.002). Participants in both the “welfare” and “normative” categories were also more likely to be missing the school completion item as compared to the ‘low’ vulnerability category (*IRR* = 0.91, 95% CI: [0.87, 0.96], *p* = 0.001 and *IRR* = 0.95, 95% CI: [0.91, 0.98], *p* = 0.007, respectively). This suggests the regression results are conservative estimates for the “high” vulnerability category in particular. Hence the relationship to early school leaving may be stronger than what has been identified in this study.

**Table 6:** Sensitivity analyses for vulnerability measures—negative binomial regression to determine missingness.

Variable	IRR	Standard error	95% CI	
			*LL*	*UL*
Vulnerability				
Low	Referent			
Normative	0.95***	0.02	0.91	0.98
Welfare	0.91***	0.02	0.87	0.96
High	0.90***	0.03	0.84	0.96
Covariates				
Cohort – middle (referent = youngest)	0.97	0.02	0.93	1.00
Cohort – oldest (referent = youngest)	0.97	0.02	0.93	1.01
Gender – female	1.08***	0.02	1.05	1.12
Student born in Australia	1.12*	0.05	1.02	1.23
Interaction with antisocial peers	0.94*	0.03	0.89	0.99
Childhood concentration/attention problems	0.98	0.01	0.96	1.01
Parent born in Australia	1.00	0.02	0.97	1.04
Suspended from school in past year	0.87*	0.05	0.78	0.98

Abbreviations: CI, confidence interval; LL, lower limit; UL, upper limit.

* *p* < 0.05;

*** *p* < 0.001.

## 6 Discussion

As educational attainment can impact work opportunities and career trajectories, and the physical and mental health of young people (Lamb 2011; Robinson and Meredith 2013), it is important to identify factors that contribute to school completion to inform efforts to improve educational equity. This study aimed to identify vulnerability indicators that predicted early school leaving to inform interventions designed to reduce school noncompletion and examined the hypothesis that early school leaving would increase as a function of the level of vulnerability (across four classes). Study findings supported this hypothesis; increasing level of vulnerability predicted an increased likelihood of early school leaving (measured 8 years later).

Results also provide some support for applying the proposed framework of child disadvantage (Goldfeld et al. 2018) to identify and operationalise indicators of vulnerability, however, they also suggest that profiles of vulnerability that predict early school leaving are defined by a combination of indicators across domains. The current study found that vulnerability was modelled by sociodemographic, geographical, and family and community risk factors across multiple ecological levels, but the categorisation differed to the Goldfeld et al. (2018) framework of child disadvantage. Despite these differences, the framework is valuable for mapping indicators to identify and prioritise vulnerable children and youth. These results highlight the importance of multidimensional measures of disadvantage and vulnerability to inform policy development and interventions aimed at improving school completion in Victoria, Australia.

The results indicated that a four‐class model (‘low,’ ‘normative,’ ‘welfare,’ and ‘high’) best characterised differing vulnerability indicator levels in this sample. Aligned with the extant literature, sociodemographic indicators of household income, main caregiver education, and welfare status appeared to be key indicators of vulnerability across groups, particularly between lower vulnerability (‘low’ and ‘normative’) and higher vulnerability groups (‘welfare’ and ‘high’) (Almquist 2016; Kallio et al. 2016; Suh et al. 2007). Family antisocial behaviour (family substance use and involvement in illegal activities), family conflict, and community disorganisation also distinguished the two higher vulnerability classes from each other. While the relationship between family risk factors and school completion aligns with prior studies (Almquist 2016; Gubbels et al. 2019), more research is needed to investigate community level risk factors on educational outcomes and how risk factors may amplify existing sociodemographic disadvantage.

The prediction of educational outcomes identified in Goldfeld et al. (2018) was replicated in the current study. Namely, youth with higher levels of vulnerability (disadvantage in Goldfeld et al. 2018) had higher subsequent levels of early school leaving (lower academic achievement in Goldfeld et al. 2018). Relative to those with sociodemographic advantage (‘low’ vulnerability), those high on sociodemographic disadvantage and risk factors (‘welfare’ and ‘high’ vulnerability), were significantly more likely to leave school before completing Year 12. Participants in the ‘high’ vulnerability and ‘welfare’ classes were characterised by the poorest educational trajectories (59% and 70% completed Year 12, respectively) as compared to the ‘normative’ and the ‘low’ vulnerability classes (80% and 95% completion). Taken together, consistent with the Goldfeld et al. (2018) framework, results suggest that factors that contribute to vulnerability tend to exist across multiple ecological levels, increasing the additive risk for early school leaving (Atkinson et al. 2015; Kallio et al. 2016; Schoon and Melis 2019).

The current findings assist identification of sociodemographic disadvantage indicators (e.g., welfare involvement) that predict adverse life course outcomes (Commission on Social Determinants of Health 2008). Findings also align with international evidence on risk accumulation across ecological levels (individual, family, and community) and context (specifically student) (Gubbels et al. 2019; Lamb 2011) and provide support for notions of cumulative disadvantage (Atkinson et al. 2015; Schoon and Melis 2019). This suggests successful methods to improve educational outcomes should target multiple domains across ecological levels and demonstrates that youth at risk of early school leaving can be identified by indicators from individual, family, and community environments during childhood and early adolescence. It is also important to note that while sociodemographic factors at the school level were not included in our measure of vulnerability, it is important to consider school‐level factors such as poverty level and exclusion practices (e.g., suspensions and expulsions) as these may also affect early school leaving (Fasang et al. 2014). Aligned with literature on social determinants, vulnerability exists on a continuum, with those affected by higher levels of vulnerability experiencing poorer educational outcomes (Commission on Social Determinants of Health 2008; Goldfeld et al. 2018).

Understanding profiles of vulnerability and disadvantage is an important step to tailor interventions, however research is also needed to investigate specific factors that can disrupt this association, as sociodemographic factors driving vulnerability are difficult to modify (i.e., parental income, education, employment, and welfare) (Schoon and Melis 2019). Identification of protective factors (e.g., opportunities and rewards for prosocial involvement) that can be strengthened to buffer the effects of sociodemographic disadvantage (Toumbourou et al. 2015) is an important next research step to improve educational equity for vulnerable youth.

### 6.1 Strengths and Limitations

The longitudinal design of the IYDS enabled prospective assessment of multiple sociodemographic and risk factors across a range of ecological levels (McMorris et al. 2007). In addition, validated scales were used to model the relationship between vulnerability and early school leaving, with adjustment for key covariates. This enabled the unique effect of multidimensional vulnerability on early school leaving to be measured. Given findings are aligned with recent research (Goldfeld et al. 2018) they are likely to translate to pre‐COVID‐19 cohorts.

There are however some limitations. First, the IYDS youth survey relied on youth‐ and parent‐report indicators and are therefore subject to self‐report bias. In addition, attrition based on vulnerability is evident with sensitivity analyses suggesting prevalence rates may be an underestimation, and the estimates may be conservative. Nevertheless, the findings highlight the value of these multidimensional indicators in forecasting groups at higher risk of not competing secondary school. The low alpha of 0.55 for the vulnerability measure may also be considered a limitation. However, as the items were drawn from diverse domains (sociodemographic, geographical, risk factors), variability across the measure was expected. The low internal consistency for this measure supports the analytic approach of identifying latent classes from vulnerability items. Second, our early school leaving outcome did not reflect nonschool education or training or capture potential benefits afforded to those who engage in further education or training after leaving school early. Future studies could incorporate measures of other types of educational attainment to gain a more nuanced understanding of vulnerability and educational outcomes, and later employment opportunities.

In addition, vulnerability indicators in the current study were measured cross‐sectionally at Wave 1 and thus provided a snapshot of vulnerability in a single year and did not capture change in vulnerability over the adolescent years. Mapping the trajectory of vulnerability over time, in relation to early school leaving, will provide a more comprehensive understanding of the pattern of this relationship, and may highlight important periods of higher vulnerability for future intervention, such as during transitional periods (e.g., primary school to high school).

Finally, sensitivity analyses suggest there were some differences between the cohort of students who participated in the IYDS and the school‐aged population in Victoria, suggesting the IYDS sample was a lower risk sample relative to the index population. The IYDS sample reported a higher rate of low‐income assistance, were more likely to live in a regional area, and reported a lower rate of early school leaving than youth in Victoria at this time. In addition, the findings in this study relate to outcomes before 2020, therefore there may be changes to vulnerability and school completion, because of the COVID‐19 pandemic, that are not reflected in these results.

### 6.2 Implications

Although existing reviews suggest there are promising programs to prevent early school leaving (Hahn et al. 2015; Rodríguez and Conchas 2009; Wilson et al. 2011), the current findings assist in identifying how to refine these programs for vulnerable youth. Existing interventions currently target single risk factors, rather than addressing the multidimensional nature of vulnerability to early school leaving (Wilson et al. 2011). It is also clear that interventions need to consider the various contexts of a child's environment rather than focusing on a single area, such as the school level. These findings confer the potential for community coalitions to use multiple indicators, guided by the Goldfeld et al. (2018) framework, to trial novel interventions within the Communities That Care (CTC) coalition model. This model seeks to address youth antisocial behaviours through identifying risk and protective factors in the environments of youth and implementing evidence‐based programs to tackle these issues, with some evidence suggesting the implementation of the CTC process reduces youth crime in Australian communities (Rowland et al. 2022). Using the risk and protective framework CTC coalitions identify and implement evidence‐based programs to improve educational outcomes, such as Classwide Peer Tutoring and the You Can Do It! program (Ashdown and Bernard 2012; Wills et al. 2025). Application of these findings within the CTC model may be an effective avenue for testing current programs and evaluating new programs to improve educational equity.

## 7 Conclusion

Experiencing high levels of family and community risk, and welfare involvement, is associated with increased vulnerability for early school leaving, 8 years later. This study identified multidimensional indicators that could profile groups vulnerable for early school leaving and provides insight into the unique contribution of risk and sociodemographic factors. The findings also contribute to community coalition efforts to improve educational equity. Prioritising interventions targeted to the structural and associated risk factors underpinning vulnerability, will likely shift educational trajectories and improve social equity in children and into the next generation.

## Author Contributions

Heidi M. Renner conceived of the study, participated in its design, performed the statistical analysis, and drafted the manuscript. Bosco Rowland and John W. Toumbourou participated in the design and interpretation of the data, and revised for intellectual content. Delyse Hutchinson participated in the design and revised for intellectual content. All authors read and approved the final manuscript. The views expressed in this paper are those of the authors and may not reflect those of their organisational affiliations, nor of other collaborating individuals or organisations.

## Ethics Statement

Ethics approval for the Australian arm of the original study was obtained from the Royal Children's Hospital Ethics in Human Research Committee (Ref: 060045X). Approval for secondary data analysis for this study and an ethics waiver was provided by Deakin University Human Research Ethics Committee (Ref: 2021‐316). All procedures performed in studies involving human participants were in accordance with the ethical standards of the institutional and/or national research committee and with the 1964 Helsinki Declaration and its later amendments or comparable ethical standards.

## Consent

Written informed consent was initially obtained from all parents before the first wave of data collection. Individual participants also provided consent upon participation at each wave of the study. At each wave of the study consent was provided for publication of the deidentified results.

## Conflicts of Interest

Bosco Rowland and John W. Toumbourou are unpaid board members with Communities That Care Ltd (CEO and director; respectively). The other authors declare no conflicts of interest.

## Supporting information

Appendix Vulnerability Predicting Education Resubmit 20250417.

## Data Availability

The International Youth Development Study dataset analysed for the current study can be accessed by request through the LifeCourse Initiative at the Murdoch Children's Research Institute, Royal Children's Hospital, Melbourne, Australia.

## References

[jad70042-bib-0001] Adhikari, P. 2006. Socio‐Economic Indexes for Areas: Introduction, Use and Future Directions (Catalogue No. 1351.0.55.015). Australian Bureau of Statistics.

[jad70042-bib-0002] Allee‐Herndon, K. A. , and S. K. Roberts . 2019. “Poverty, Self‐Regulation and Executive Function, and Learning in k‐2 Classrooms: A Systematic Literature Review of Current Empirical Research.” Journal of Research in Childhood Education 33, no. 3: 345–362. 10.1080/02568543.2019.1613273.

[jad70042-bib-0003] Almquist, Y. B. 2016. “Childhood Origins and Adult Destinations: The Impact of Childhood Living Conditions on Coexisting Disadvantages in Adulthood.” International Journal of Social Welfare 25, no. 2: 176–186. 10.1111/ijsw.12178.

[jad70042-bib-0004] Arthur, M. W. , J. D. Hawkins , J. A. Pollard , R. F. Catalano , and Jr Baglioni AJ, Jr. 2002. “Measuring Risk and Protective Factors for Substance Use, Delinquency, and Other Adolescent Problem Behaviors: The Communities That Care Youth Survey.” Evaluation Review 26, no. 6: 575–601. 10.1177/019384102237850. 12465571

[jad70042-bib-0005] Ashdown, D. M. , and M. E. Bernard . 2012. “Can Explicit Instruction in Social and Emotional Learning Skills Benefit the Social‐Emotional Development, Well‐Being, and Academic Achievement of Young Children?” Early Childhood Education Journal 39: 397–405. 10.1007/s10643-011-0481-x.

[jad70042-bib-0006] Atkinson, L. , J. Beitchman , A. Gonzalez , et al. 2015. “Cumulative Risk, Cumulative Outcome: A 20‐Year Longitudinal Study.” PLoS One 10, no. 6: e0127650. 10.1371/journal.pone.0127650. 26030616 PMC4452593

[jad70042-bib-0007] Australian Bureau of Statistics [ABS] . 2009. *Education and Work (Catalogue No*. *6227.0)* . Australin Bureau of Statistics, Commonwealth of Australia. https://www.ausstats.abs.gov.au/ausstats/subscriber.nsf/0/92DCF2C65BFBF519CA257677001486D9/$File/62270_May%202009_Reissue.pdf.

[jad70042-bib-0008] Australian Curriculum Assessment and Reporting Authority . 2025. National Report on Schooling in Australia: Year 12 Certification Rates. Australian Curriculum, Assessment and Reporting Authority. https://www.acara.edu.au/reporting/national-report-on-schooling-in-australia/year-12-certification-rates.

[jad70042-bib-0009] Barker, B. , T. Kerr , H. Dong , E. Wood , and K. DeBeck . 2017. “High School Incompletion and Childhood Maltreatment Among Street‐Involved Young People in Vancouver, Canada.” Health & Social Care in the Community 25, no. 2: 378–384. 10.1111/hsc.12314. 26709010 PMC5037052

[jad70042-bib-0010] Bond, L. , H. Butler , L. Thomas , et al. 2007. “Social and School Connectedness In Early Secondary School as Predictors of Late Teenage Substance Use, Mental Health, and Academic Outcomes.” Journal of Adolescent Health 40, no. 4: 357.e9–357.e18. 10.1016/j.jadohealth.2006.10.013. 17367730

[jad70042-bib-0011] Bowman, S. , C. McKinstry , and P. McGorry . 2017. “Youth Mental Ill Health and Secondary School Completion in Australia: Time to Act.” Early Intervention in Psychiatry 11, no. 4: 277–289. 10.1111/eip.12357. 27381567

[jad70042-bib-0012] Bronfenbrenner, U. , and P. A. Morris . 2006. “The Bioecological Model of Human Development.” In Handbook of Child Psychologyedited by W. Damon and R. M. Lerner , 793–828. John Wiley & Sons.

[jad70042-bib-0013] Chau, K. , C. Schweitzer‐Troester , B. Leroy , and B. Kabuth . 2022. “Associations Between School Difficulties and Family Type and the Role of Socioeconomic, Behavior and Health‐Related Difficulties in Early Adolescents: A Population‐Based Study.” Nordic Journal of Psychiatry 76, no. 8: 623–633. 10.1080/08039488.2022.2030402. 35112630

[jad70042-bib-0014] Commission on Social Determinants of Health 2008. Closing the Gap in a Generation: Health Equity Through Action on the Social Determinants of Health. Final Report of the Commission on Social Determinants of Health. World Health Organization.

[jad70042-bib-0015] Doll, J. J. , Z. Eslami , and L. Walters . 2013. “Understanding Why Students Drop out of High School, According to Their Own Reports: Are They Pushed or Pulled, or Do They Fall Out? A Comparative Analysis of Seven Nationally Representative Studies.” Sage Open 3, no. 4: 1–15. 10.1177/2158244013503834.

[jad70042-bib-0016] Fasang, A. E. , W. Mangino , and H. Brückner . 2014. “Social Closure and Educational Attainment.” Sociological Forum 29, no. 1: 137–164. 10.1111/socf.12073.

[jad70042-bib-0017] Feinberg, M. E. , D. Jones , M. T. Greenberg , D. W. Osgood , and D. Bontempo . 2010. “Effects of the Communities That Care Model in Pennsylvania on Change in Adolescent Risk and Problem Behaviors.” Prevention Science 11, no. 2: 163–171. 10.1007/s11121-009-0161-x. 20020209 PMC4454391

[jad70042-bib-0018] Goldfeld, S. , M. O'Connor , D. Cloney , et al. 2018. “Understanding Child Disadvantage From a Social Determinants Perspective.” Journal of Epidemiology and Community Health 72, no. 3: 223–229. 10.1136/jech-2017-209036. 29263179

[jad70042-bib-0019] Goza, F. , and I. Ryabov . 2009. “Adolescents' Educational Outcomes: Racial and Ethnic Variations in Peer Network Importance.” Journal of Youth and Adolescence 38, no. 9: 1264–1279. 10.1007/s10964-009-9418-8. 19669905 PMC3552487

[jad70042-bib-0020] Gubbels, J. , C. E. van der Put , and M. Assink . 2019. “Risk Factors for School Absenteeism and Dropout: A Meta‐Analytic Review.” Journal of Youth and Adolescence 48, no. 9: 1637–1667. 10.1007/s10964-019-01072-5. 31312979 PMC6732159

[jad70042-bib-0021] Hahn, R. A. , J. A. Knopf , S. J. Wilson , et al. 2015. “Programs to Increase High School Completion.” American Journal of Preventive Medicine 48, no. 5: 599–608. 10.1016/j.amepre.2014.12.005. 25818117 PMC4681508

[jad70042-bib-0022] Hemphill, S. A. , J. A. Heerde , T. I. Herrenkohl , G. C. Patton , J. W. Toumbourou , and R. F. Catalano . 2011. “Risk and Protective Factors for Adolescent Substance Use in Washington State, the United States and Victoria, Australia: A Longitudinal Study.” Journal of Adolescent Health 49, no. 3: 312–320. 10.1016/j.jadohealth.2010.12.017. PMC3032384 21856525

[jad70042-bib-0023] Hemphill, S. A. , J. W. Toumbourou , R. Smith , et al. 2010. “Are Rates of School Suspension Higher in Socially Disadvantaged Neighbourhoods? An Australian Study.” Health Promotion Journal of Australia 21, no. 1: 12–18. 10.1071/he10012. 20406147

[jad70042-bib-0024] Kallio, J. M. , T. M. Kauppinen , and J. Erola . 2016. “Cumulative Socio‐Economic Disadvantage and Secondary Education in Finland.” European Sociological Review 32, no. 5: 649–661. 10.1093/esr/jcw021.

[jad70042-bib-0025] Kao, G. , and J. S. Thompson . 2003. “Racial and Ethnic Stratification in Educational Achievement and Attainment.” Annual Review of Sociology 29: 417–442. 10.1146/annurev.soc.29.010202.100019.

[jad70042-bib-0026] Kitchin, J. L. , and N. J. Karlin . 2024. “The Social Ecology of Academic Achievement: Modeling Social Sources of Protection.” European Journal of Psychology of Education 39, no. 2: 475–502. 10.1007/s10212-023-00702-8.

[jad70042-bib-0027] Lamb, S. 2011. School Dropout and Completion: International Comparative Studies in Theory and Policy. Springer.

[jad70042-bib-0028] Lamb, S. , S. Huo , and A. Walstab , et al. 2020. Educational Opportunity in Australia 2020: Who Succeeds and Who Misses Out. Centre for International Research on Education Systems, Victoria University, for the Mitchell Institute.

[jad70042-bib-0029] Lanza, S. T. , B. L. Rhoades , R. L. Nix , and M. T. Greenberg . 2010. “Modeling the Interplay of Multilevel Risk Factors for Future Academic and Behavior Problems: A Person‐Centered Approach.” Development and Psychopathology 22, no. 2: 313–335. 10.1017/S0954579410000088. 20423544 PMC3005302

[jad70042-bib-0030] Marmot, M. , S. Friel , R. Bell , T. A. Houweling , and S. Taylor . 2008. “Closing the Gap in a Generation: Health Equity Through Action on the Social Determinants of Health.” Lancet 372, no. 9650: 1661–1669. 10.1016/S0140-6736(08)61690-6. 18994664

[jad70042-bib-0031] McMorris, B. J. , S. A. Hemphill , J. W. Toumbourou , R. F. Catalano , and G. C. Patton . 2007. “Prevalence of Substance Use and Delinquent Behavior in Adolescents From Victoria, Australia and Washington State, United States.” Health Education & Behavior: The Official Publication of the Society for Public Health Education 34, no. 4: 634–650. 10.1177/1090198106286272. 16740513

[jad70042-bib-0032] Muthén, L. K. , and B. O. Muthén . 1998–2017. Mplus User's Guide: Statistical Analysis With Latent Variables. Muthen & Muthen.

[jad70042-bib-0033] Rendón, M. G. 2014. “‘Caught Up’: How Urban Violence and Peer Ties Contribute to High School Noncompletion.” Social Problems 61, no. 1: 61–82. 10.1525/sp.2013.11237.

[jad70042-bib-0034] Robinson, E. , and V. Meredith . 2013. “Family Factors in Early School Leaving.” Journal of the Home Economics Institute of Australia 20, no. 2: 30–40. 10.3316/aeipt.202673.

[jad70042-bib-0035] Rodríguez, L. F. , and G. Q. Conchas . 2009. “Preventing Truancy and Dropout Among Urban Middle School Youth: Understanding Community‐Based Action From the Student's Perspective.” Education & Urban Society 41, no. 2: 216–247. 10.1177/0013124508325681.

[jad70042-bib-0036] Roos, L. L. , and E. Wall‐Wieler . 2017. “Life Course Epidemiology: Modeling Educational Attainment With Administrative Data.” PLoS One 12, no. 12: e0188976. 10.1371/journal.pone.0188976. 29281651 PMC5744927

[jad70042-bib-0037] Rowland, B. , A. B. Kelly , M. Mohebbi , et al. 2022. “Evaluation of Communities That Care—Effects on Municipal Youth Crime Rates in Victoria, Australia: 2010–2019.” Prevention Science 23: 24–35. 10.1007/s11121-021-01297-6. 34626325

[jad70042-bib-0038] Schoon, I. , and G. Melis . 2019. “Intergenerational Transmission of Family Adversity: Examining Constellations of Risk Factors.” PLoS One 14, no. 4: e0214801. 10.1371/journal.pone.0214801. 31017914 PMC6481806

[jad70042-bib-0039] Silins, E. , D. M. Fergusson , G. C. Patton , et al. 2015. “Adolescent Substance Use and Educational Attainment: An Integrative Data Analysis Comparing Cannabis and Alcohol From Three Australasian Cohorts.” Drug and Alcohol Dependence 156: 90–96. 10.1016/j.drugalcdep.2015.08.034. 26409754

[jad70042-bib-0040] StataCorp . 2021. Stata Statistical Software: Release 17. StataCorp LLC.

[jad70042-bib-0041] Suh, S. , J. Suh , and I. Houston . 2007. “Predictors of Categorical at‐Risk High School Dropouts.” Journal of Counseling & Development 85, no. 2: 196–203. 10.1002/j.1556-6678.2007.tb00463.x.

[jad70042-bib-0042] The Smith Family 2024. “Pathways, Engagement and Transitions: Experiences of Early School Leavers.” In Pathways, Engagement and Transitions Report 3. The Smith Family.

[jad70042-bib-0043] Toumbourou, J. W. , R. K. Leung , R. Homel , K. Freiberg , L. Satyen , and S. A. Hemphill . 2015. “Violence Prevention and Early Intervention: What Works?” In Preventing Violence in Australia: Policy, Practice and Solutionsedited by A. Day and E. Fernandez , 45–62. Federation Press.

[jad70042-bib-0044] Toumbourou, J. W. , B. Rowland , J. Williams , R. Smith , and G. C. Patton . 2019. “Community Intervention to Prevent Adolescent Health Behavior Problems: Evaluation of Communities That Care in Australia.” Health Psychology 38, no. 6: 536–544. 10.1037/hea0000735. 30998065

[jad70042-bib-0045] Townsend, L. , A. J. Flisher , and G. King . 2007. “A Systematic Review of the Relationship Between High School Dropout and Substance Use.” Clinical Child and Family Psychology Review 10, no. 4: 295–317. 17636403 10.1007/s10567-007-0023-7

[jad70042-bib-0046] Trewin, D. 2001. Outcomes of ABS Views on Remoteness Consultation, Australia (Catalogue No. 1244.0.00.001). Australian Bureau of Statistics, Commonwealth of Australia.

[jad70042-bib-0047] Wake, M. , S. Goldfeld , and A. Davidson . 2022. “Embedding Life Course Interventions in Longitudinal Cohort Studies: Australia's GenV Opportunity.” Pediatrics 149, no. Suppl 5: e2021053509R. 10.1542/peds.2021-053509R. PMC9847412 35503324

[jad70042-bib-0048] Weber, S. , N. Kronberger , and M. Appel . 2018. “Immigrant Students' Educational Trajectories: The Influence of Cultural Identity and Stereotype Threat.” Self & Identity 17, no. 2: 211–235. 10.1080/15298868.2017.1380696.

[jad70042-bib-0049] Wills, H. P. , D. Kamps , and C. R. Greenwood . 2025. “Peer Tutoring and Teams Interventions for School‐Aged Students: CWPT and CW‐FIT.” Education and Treatment of Children. 10.1007/s43494-025-00151-6.

[jad70042-bib-0050] Wilson, S. J. , and E. E. Tanner‐Smith . 2013. “Dropout Prevention and Intervention Programs for Improving School Completion Among School‐Aged Children and Youth: A Systematic Review.” Journal of the Society for Social Work & Research 4, no. 4: 357–372. 10.5243/jsswr.2013.22.

[jad70042-bib-0051] Wilson, S. J. , E. E. Tanner‐Smith , M. W. Lipsey , K. Steinka‐Fry , and J. Morrison . 2011. “Dropout Prevention and Intervention Programs: Effects on School Completion and Dropout Among School‐Aged Children and Youth.” Campbell Systematic Reviews 7, no. 1: 1–61. 10.4073/csr.2011.8.

