# Supplementary material for: Profiling Vulnerability in Youth and Predicting Educational Attainment in Young Adulthood
Source: J Adolesc. 2025 Aug 18;98(1):82–94. doi: 10.1002/jad.70042 (PMC12780652; doi:10.1002/jad.70042)
Supplement: Supplementary file 1 — Appendix Vulnerability Predicting Education Resubmit 20250417. [file JAD-98-82-s001.docx]

**Supplementary File**

**Appendix A**

*Model Alignment between the Child Disadvantage Framework (Goldfeld et al., 2018) and the IYDS*

| Child disadvantage framework (Goldfeld et al., 2018) | International Youth Development Study (IYDS) measure |
| --- | --- |
| Sociodemographic domain | |
| Child speaks language other than English | X |
| Child has medical condition or disability | X |
| Main caregiver income | X |
| Household income | Annual pre-tax household income ^a^ |
| Main caregiver speaks language other than English | Family speaks language other than English ^a^ |
| Main caregiver level of education | Main caregiver highest level of education ^a^ |
| Main caregiver occupation | Main caregiver employment status ^a^ |
| Main caregiver financial hardship | Main caregiver/partner recipient of welfare ^a^ |
| Parents of child are partners | Parents of child are partners (New) ^a^ |
| Number of people in household | Number of people in household ^a^ |
| Geographical environments domain | |
| Neighbourhood liveability | Community disorganisation ^b^ |
| Community socioeconomic status | Community SES (New) ^c^ |
| Urban versus regional location | Lives in a regional area (New) ^c^ |
| Health conditions domain | |
| Child tooth decay | X |
| Main caregiver depression | X |
| Main caregiver medical condition or disability | X |
| Risk factors domain | |
| Child body mass index | X |
| Main caregiver body mass index | X |
| Child eats high fat foods and high sugar drinks | X |
| Main caregiver smoking status | Family history of antisocial behaviour ^b^ |
| Main caregiver binge drinking | Family history of antisocial behaviour ^b^ |
| Child unmet need for services | X |
| Main caregiver physical arguments with partner | Family conflict ^b^ |
| Home education environment | X |
| Number of homes child lived in | Number of times changed homes since kindergarten ^b^ |
| Child physical inactivity | X |
| Main caregiver argumentative partner relationship | Family conflict ^b^ |
| Main caregiver angry parenting style | Family conflict ^b^ |
| Stressful life events within the family | Family conflict ^b^ |
| Main caregiver unmet need for social support | X |
| *Note*. ^a^ = parent-report indicator; ^b^ = student/youth-report indicator. ^c^ = geographic indicator coded from home location. | |

**Appendix B**

*IYDS Indicator Detail for Vulnerability, Early School Leaving, and Covariates*

| Indicator | Description | # items |
| --- | --- | --- |
| Vulnerability | | |
| Sociodemographic | | |
| 1. Annual pre-tax household income | Measure of annual pre-tax household income combining two items to create one variable, coded with 19 categories. An increase in the score reflects an increase in income level.  *Example question: “*What is your family’s combined yearly income before taxes? Is it under $30,000 or above $30,000?”; with four response options: (1) $30,000 or under, (2) Above $30,000, (3) Refused, and (4) Don’t know. Followed by: *“*Would that be…”; with 16 options: (1) $30,001 to $40,000, (2) $40,001 to $50,000, (3) $50,001 to $60,000, (4) $60,001 to $70,000, (5) $70,001 to $80,000, (6) $80,001 to $90,000, (7) $90,001 to $100,000, (8) $100,001 to $110,000, (9) $110,001 to $120,000, (10) $120,001 to $130,000, (11) $130,001 to $140,000, (12) $140,001 to $150,000, (13) $150,001 to $200,000, (14) $200,001 and over, (15) Refused, (16) Don't know. Recoded to 19 income brackets categories. | 1 |
| 1. Family speaks language other than English | Measure of language spoken in the home. An increase in the score reflects a language other than English is spoken in the family (recoded).  *Question wording: “*Does your family primarily speak a language other than English at home?”; with four response options: (1) Yes, (2) No, (3) Refused, (4) Don’t know. | 1 |
| 1. Main caregiver completed secondary school | Measure of parental educational achievement created by collapsing 13 categories to four. An increase in the score reflects the main caregiver completed secondary school.  *Question wording:* “What’s your highest level of education?”; with 13 response options: (1) Less than Year 7, (2) Year 7, (3) Year 8, (4) Year 9, (5) Year 10, (6) Year 11, (7) Year 12, (8) TAFE (Technical And Further Education) certificate/apprenticeship, (9) Some university, (10) 3 year university degree, (11) 4 year university degree, (12) postgraduate, (13) other.  Collapsed into four categories: (1) Less than secondary school (year/ grade 11 or less), (2) Completed secondary school (Year 12, TAFE certificate/apprenticeship, some university), (3) Completed post secondary education (3 or 4 year university degree, postgraduate degree), and (4) Other. Recoded to a binary variable to reflect whether the main caregiver completed secondary school or not (no/yes). | 1 |
| 1. Main caregiver in paid work | Measure of employment status created by collapsing eight categories into two. An increase in the score reflects the main caregiver is in paid work (employed).  *Question wording:* “Employment Status”; with eight response options: (1) In paid work, (2) On leave from paid work, (3) Unemployed looking for work, (4) Unemployed and working for the dole, (5) Student, (6) Retired, (7) Not working but not retired and not looking for other work, and (8) Other.  Collapsed into two categories: (1) Not in Paid Work (unemployed looking for work, student, retired, not working but not retired and not looking for other work, other), and (2) In Paid Work (in paid work, on leave from paid work, unemployed and working for the dole). | 1 |
| 1. Main caregiver/partner recipient of welfare | Measure of low-income status. An increase in the score reflects the main caregiver was the recipient of welfare in the past year (recoded).  *Question wording:* “In the past year, have you or your partner had a health care concession card?”; with two response options: (1) Yes, and (2) No. | 1 |
|  |  |  |
| Indicator | Description | # items |
| 1. Biological parents of child are partners (New) | A new indicator of family structure. An increase in the score reflects the biological parents of the student were partners.  Created by combining the following two items into a new indicator with two categories (no/yes).  *Question wording: “*Current marital status”; with nine response options: (1) Single, having never been married, (2) Married, (3) Living with partner, (4) Widowed, (5) Divorced, (6) Separated but not divorced, (7) Other (specify), (8). Refused, (9) Don’t know.  *Question wording: “*What is your spouse’s relationship to the student?”; with 16 response options: (1) Natural Mother, (2) Natural Father, (3) Stepmother, (4) Stepfather, (5) Adoptive Mother, (6) Adoptive Father, (7) Foster Mother, (8) Foster Father, (9) Live-in Partner of Parent, (10) Grandmother, (11) Grandfather, (12) Other Relative, (13) Other Unrelated Adult, (14) Other (specify), (15) Refused, (16) Don't Know. | 2 |
| 1. Number of people in household | Measure of household size. An increase in the score reflects larger households.  *Question wording:* “How many people currently live in your household? (Please include anyone who may have a residence elsewhere but stays there most of the time).”; with a numeric response provided. Original categories maintained. | 1 |
| Geographical environments | | |
| 1. Community disorganisation | Validated risk factor scale that captures high population density, physical deterioration, and rates of adult crime, and juvenile crime and drug use within the community (Arthur et al., 2002). An increase in the score reflects higher levels of community disorganisation.  *Example question:* “How much do each of the following statements describe your neighbourhood: I feel safe in my neighbourhood.”; with four response options: (1) NO!, (2) no, (3), yes, and (4) YES!. | 5 |
| 1. Community SES (New) | A new indicator created to capture community-level socio-economic status. An increase in the score reflects higher community SES.  Created based on the Index of Relative Socio-Economic Advantage / Disadvantage (IRSEAD) from the Australian Bureau of Statistics (ABS). IRSEAD was identified using the reported postcode of the student’s home. IRSEAD was reported as a numeric response for each postcode. | 1 |
| 1. Lives in a regional area (New) | A new indicator created to capture urban versus regional location. An increase in the score reflects that the student lives in a regional area.  Created using the ABS definition for remoteness with six response options: (1) major cities, (2) inner regional, (3) outer regional, (4) remote, (5) very remote, and (6) migratory. The six categories were collapsed into a binary indicator (no/yes). | 1 |
| Risk factors | | |
| 1. Family history of antisocial behaviour | Validated risk factor scale that captures the risk associated with being born or raised in a family with a history of alcoholism (Arthur et al., 2002). An increase in the score reflects higher rates of family antisocial behaviours.  *Example question:* “Have any of your brothers of sisters ever: been suspended or expelled from school?”; with three response options (1) No, (2) Yes, and (3) I don’t have any brothers or sisters. | 10 |
| 1. Family conflict | Validated risk factor scale that captures the risk associated with being raised in a family high in conflict. An increase in the score reflects higher rates of family conflict.  *Example question:* “We argue about the same things in my family over and over.”; with four response options: (1) NO!, (2) no, (3), yes, and (4) YES!. | 3 |
|  |  |  |
| Indicator | Description | # items |
| 1. Number of times changed homes since kindergarten | Single item to capture transitions and mobility. An increase in the score reflects higher number of times the student has moved homes.  *Question wording:* “How many times have you changed **homes** since  kindergarten?”; with five response options: (1) Never, (2) 1 or 2 times, (3) 3 or 4 times, (4) 5 or 6 times, (5) 7 or more times. | 1 |
| Early school leaving | | |
| 1. Highest year of secondary school completed | Single item to capture early school leaving. An increase in the score reflects earlier school leaving (i.e., leaving school at a younger age/year level).  *Question wording:* “What was the highest year level at secondary school you completed?”; with six response options: (1) Year 12 or equivalent, (2) Year 11 or equivalent, (3) Year 10 or equivalent, (4) Year 9 or equivalent, (5) Year 8 or below, and (6) Other, with a text response to capture ‘Other’. | 1 |
| Covariates | | |
| Demographic |  |  |
| 1. Gender | Participant gender was derived from school enrolment records. Two response options were included: (1) Male and (2) Female. An increase in the score reflects female. | 1 |
| 1. Study cohort | Three cohorts were included: (1) youngest (Grade 5; primary school), (2) middle (Year 7; early secondary school), and (3) oldest (Year 9; mid-secondary school). An increase in the score reflects a higher year level. | 1 |
| Childhood emotional & behavioural problems | | |
| 1. Sensation seeking | Validated risk factor scale that captures the risk associated with engaging in risky and thrilling behaviours. An increase in the score reflects higher levels of sensation seeking behaviours.  *Example question:* “How many times have you…Done crazy things even if they are a little dangerous.”; with six response options: (1) Never, (2) I’ve done it, but not in the past year, (3) Less than once a month, (4) About once a month, (5) 2 or 3 times a month, and (6) Once a week or more. | 3 |
| 1. Rebelliousness | Validated risk factor scale that captures the risk associated with young people who take an active rebellious stance to society. An increase in the score reflects an increase in rebellious behaviours.  *Example question:* “I do the opposite of what people tell me, just to get them mad.”; with four response options: (1) NO!, (2) no, (3), yes, and (4) YES!. | 3 |
| 1. Childhood impulsivity | Validated risk factor scale that captures the risk associated with a tendency to act impulsively. An increase in the score reflects an increase in impulsive behaviours.  *Example question:* “I rush into things, starting before I know what to do.”; with four response options: (1) NO!, (2) no, (3), yes, and (4) YES!. | 3 |
| 1. Childhood concentration/attention | Validated risk factor scale that captures the risk associated with behavioural problems with attention and concentration. An increase in the score reflects and increase in difficulties concentrating.  *Example question:* “I find it hard to keep concentrating on tasks.”; with four response options: (1) NO!, (2) no, (3), yes, and (4) YES!. | 2 |
| Mental health problems |  |  |
| 1. Depression symptomology | Validated depression scale from the Short Mood and Feelings Questionnaire (Angold et al., 1995). An increase in the score reflects an increase in reported depressive symptomology.  *Example question:* “Please indicate how true each of the following statements has been for you during the last 30 days (1 month)…I felt miserable or unhappy.”; with three response options: (1) Not True, (2) Sometimes True, and (3) True. | 13 |
| Indicator | Description | # items |
| Antisocial behaviour |  |  |
| 1. Interaction with antisocial peers | Validated risk factor scale that captures the risk associated with connection to antisocial peers who engage in delinquent or violent behaviours. An increase in the score reflects socialisation and connection to a higher number of antisocial peers, and therefore an increasing risk.  *Example question:* “In the past year (12 months), how many of your best friends have… dropped out of school?”; with five response options: (1) None of my friends, (2) 1 of my friends, (3) 2 of my friends, (4) 3 of my friends, and (5) 4 of my friends. | 8 |
| 1. Favourable attitudes towards antisocial behaviour | Validated risk factor scale that captures the risk associated with positive attitudes towards delinquency and violence. An increase in the score reflects more positive attitudes towards antisocial behaviours.  *Example question:* “How wrong do you think it is for someone your age to… pick a fight with someone?”; with four response options: (1) Very Wrong, (2) Wrong, (3) A Little Bit Wrong, and (4) Not Wrong at All. | 3 |
| 1. Rewards for antisocial behaviour | Validated risk factor scale that captures the risk associated with the perception that antisocial behaviour will lead to rewards. An increase in the score reflects an increase in the belief that antisocial behaviour will be rewarded.  *Example question:* “What are the chances you would be seen as cool if you…Smoked cigarettes?”; with five response options: (1) No or Very Little Chance, (2) Little Chance, (3) Some Chance, (4) Pretty Good Chance, and (5) Very Good Chance. | 4 |
| 1. Relational aggression | Two item scale to capture the frequency of aggression towards others, both verbal and physical. An increase in the score reflects an increase in the frequency of relational aggression.  *Example question:* “How many times in the past year (12 months) have you… gotten back at another student by not letting them be in your group of friends?”; with eight response options: (1) Never, (2) 1 or 2 times, (3) 3 to 5 times, (4) 6 to 9 times, (5) 10 to 19 times, (6) 20 to 29, (7) 30 to 39, and (8) 40+ times. | 2 |
| Substance use |  |  |
| 1. Friend’s use of drugs | Validated risk factor scale that captures the risk associated with connection to peers who engage in high levels of alcohol or substance use. An increase in the score reflects higher numbers of friends who engage in alcohol or substance use.  *Example question:* “In the past year (12 months), how many of your best friends have… tried alcohol (like beer, wine or liquor/spirits) when their parents didn't know about it?”; with five response options: (1) None of my friends, (2) 1 of my friends, (3) 2 of my friends, (4) 3 of my friends, and (5) 4 of my friends. | 4 |
| 1. Perceived risks of drug use | Validated risk factor scale that captures the risk associated with whether young people perceive substance use to be risky. An increase in the score reflects an increase in the perception that drug use is not dangerous.  *Example question:* “How much do you think people risk harming themselves (physically or in other ways) if they…Use marijuana (pot, weed, grass) regularly?”; with four response options: (1) No risk, (2) Slight Risk, (3) Moderate Risk, and (4) Great Risk. | 4 |
| 1. Laws and norms favourable to drug use | Validated risk factor scale that captures the risk associated with living in a community where normative attitudes suggests substance use is acceptable for young people, and where laws regulating substance use are poorly enforced. An increase in the score reflects higher levels of permissive attitudes in the community towards substance use by minors.  *Example question:* “If a kid drank some alcohol (like beer, wine or liquor/spirits) in your neighbourhood would he or she be caught by the police?”; with four response options: (1) NO!, (2) no, (3), yes, and (4) YES!. | 6 |

| Indicator | Description | # items |
| --- | --- | --- |
| Ethnicity factors |  |  |
| 1. Parent ethnic background | Single item to capture parental ethnic background as an indicator of the student ethnicity. An increase in the score reflects the parent did not have an Australian ethnic background.  *Question wording:* “Of the following, what race or ethnicity do you consider yourself to be?”; with nine response options: (1) Australian, (2) African, (3) Asian, (4) Spanish, (5) Aboriginal, (6) Torres Strait Islander, (7) Pacific Islander, (8) Mixed ethnicity, and (9) Other.  These nine categories were collapsed into two: (1) Australian ethnicity (including Aboriginal and Torres Strait Islander) and (2) non-Australian. | 1 |
| 1. Parent born in Australia | Single item to capture parental country of birth. An increase in the score reflects the parent was born in a country other than Australia.  *Question wording:* “Where were you born?”; with 19 response options: (1) Australia, (2) New Zealand, (3) UK, (4) Italy, (5) Greece, (6) China, (7) Lebanon, (8) Vietnam, (9) Germany, (10) Spain, (11) Macedonia, (12) Phillipines, (13) Croatia, (14) Poland, (15) Malta, (16) Turkey, (17) Netherlands, (18) France, and (19) Other (record). | 1 |
| 1. Student born in Australia | Single item to capture the student’s country of birth. An increase in the score reflects the student was born in a country other than Australia.  *Question wording:* “In what country was the student born?”; with 19 response options: (1) Australia, (2) New Zealand, (3) UK, (4) Italy, (5) Greece, (6) China, (7) Lebanon, (8) Vietnam, (9) Germany, (10) Spain, (11) Macedonia, (12) Phillipines, (13) Croatia, (14) Poland, (15) Malta, (16) Turkey, (17) Netherlands, (18) France, and (19) Other (record). | 1 |
| Parenting problems |  |  |
| 1. Poor family management | Validated risk factor scale that captures the risk associated with a variety of family practices, such as unclear expectations, low monitoring, inconsistent and/or unusually harsh punishment. An increase in the score reflects poorer family management practices.  *Example question:* “Would your parents know if you did not come home on time?”; with four response options: (1) NO!, (2) no, (3), yes, and (4) YES!. | 9 |
| Problems with or at school |  |  |
| 1. Low commitment to school | Validated risk factor scale that captures the risk associated with low engagement with school, such as not liking school, not completing homework, and not seeing value in education. An increase in the score reflects an increase in low commitment, or a decrease in commitment, to school.  *Example question:* “During the last four weeks how many whole days have you missed because you skipped or “cut/wagged”?”; with seven response options: (1) None, (2) 1, (3) 2, (4) 3, (5) 4-5, (6) 6-10, and (7) 11 or more. | 7 |
| 1. Suspended from school in past year | Single item to capture the student’s past year suspension. An increase in the score reflects an increase in the frequency of suspensions.  *Question wording:* “How many times in the past year (12 months) have you…been suspended from school?”; with eight response options: (1) Never, (2) 1 or 2 times, (3) 3 to 5 times, (4) 6 to 9 times, (5) 10 to 19 times, (6) 20 to 29, (7) 30 to 39, and (8) 40+ times. | 1 |
| *Note.* Taken from the IYDS student survey, CTC Youth Survey Scale Dictionary (Glaser et al., 2005), and CTC survey development (Arthur et al., 2002). The numbering of the response options is for clarity and does not represent the coding of these items. | | |

**Appendix C**

*Vulnerability Indicator Correlation Matrix (N = 2,884)*

|  | 1. | 2. | 3. | 4. | 5. | 6. | 7. | 8. | 9. | 10. | 11. | 12. | 13. | |
| --- | --- | --- | --- | --- | --- | --- | --- | --- | --- | --- | --- | --- | --- | --- |
| 1. Income | - |  |  |  |  |  |  |  |  |  |  |  |  | |
| 2. Household size | 0.15*** | - |  |  |  |  |  |  |  |  |  |  |  | |
| 3. Speaks English | -0.10*** | 0.04* | - |  |  |  |  |  |  |  |  |  |  | |
| 4. Employed | 0.28*** | -0.11*** | -0.07*** | - |  |  |  |  |  |  |  |  |  | |
| 5. Welfare recipient | -0.61*** | -0.07*** | 0.09*** | -0.32*** | - |  |  |  |  |  |  |  |  | |
| 6. Parents are partners | 0.07** | -0.03 | 0.05* | 0.04 | -0.13*** | - |  |  |  |  |  |  |  | |
| 7. Parent education | 0.29*** | 0.01 | 0.07*** | 0.17*** | -0.19*** | 0.06** | - |  |  |  |  |  |  | |
| 8. Community disorganisation | -0.12*** | -0.05** | 0.004 | -0.06** | 0.10*** | -0.02 | -0.07*** | - |  |  |  |  |  | |
| 9. Community SES | 0.35*** | 0.01 | -0.04* | 0.10*** | -0.23*** | 0.08*** | 0.23*** | -0.09*** | - |  |  |  |  | |
| 10. Lives in regional area | -0.13*** | 0.001 | -0.24*** | 0.03 | 0.09*** | -0.05* | -0.11*** | -0.08*** | -0.42*** | - |  |  |  | |
| 11. Number times moved | -0.10*** | -0.06*** | 0.04* | -0.06** | 0.17*** | -0.30*** | -0.02 | 0.05** | -0.02 | 0.02 | - |  |  | |
| 12. Family antisocial behaviour | -0.14*** | -0.05* | -0.06*** | -0.03 | 0.13*** | -0.12*** | -0.12*** | 0.31*** | -0.11*** | 0.11*** | 0.11*** | - |  | |
| 13. Family conflict | -0.07** | 0.002 | -0.004 | -0.02 | 0.05** | -0.07** | -0.06** | 0.28*** | 0.02 | -0.002 | 0.10*** | 0.34*** | - | |
| *Note.* * *p* < .05, ** *p* < .01, *** *p* < .001. | | | | | | | | | | | | | |  |

**Appendix D**

*List of Covariates Included in Negative Binomial Regression across Eight Key Areas from Proximal (Peer-Individual) to Distal (Community) Domains*

| Variable | Domain | Entered model | Result |
| --- | --- | --- | --- |
| Demographic | | | |
| Gender | Peer-individual | Step 1 | Non-sig at step 3 |
| Study cohort ^a^ | Peer-individual | Step 1 | Significant |
| Childhood emotional & behavioural problems | | | |
| Sensation seeking | Peer-individual | Step 1 | Non-sig at step 1 |
| Rebelliousness | Peer-individual | Step 1 | Non-sig at step 1 |
| Childhood impulsivity | Peer-individual | Step 1 | Non-sig at step 1 |
| Childhood concentration/attention ^a^ | Peer-individual | Step 1 | Significant |
| Mental health problems | | | |
| Outcome: new depression | Peer-individual | Step 1 | Non-sig at step 1 |
| Antisocial behaviour | | | |
| Interaction with antisocial peers ^a^ | Peer-individual | Step 1 | Significant |
| Favorable attitudes towards antisocial behaviour | Peer-individual | Step 1 | Non-sig at step 1 |
| Rewards for antisocial involvement | Peer-individual | Step 1 | Non-sig at step 1 |
| Relational aggression - past year | Peer-individual | Step 1 | Non-sig at step 1 |
| Substance use | | | |
| Friend’s use of drugs | Peer-individual | Step 1 | Non-sig at step 1 |
| Perceived risk of drug use | Peer-individual | Step 1 | Non-sig at step 1 |
| Laws and norms favourable to drug use | Community | Step 4 | Non-sig at step 4 |
| Ethnicity factors | | | |
| Parent ethnic background | Family | Step 2 | Non-sig at step 2 |
| Parent born in Australia ^a^ | Family | Step 2 | Significant |
| Student born in Australia ^a^ | Family | Step 2 | Significant |
| Parenting problems | | | |
| Poor family management | Family | Step 2 | Non-sig at step 2 |
| Problems with or at school | | | |
| Low commitment to school | School | Step 3 | Non-sig at step 3 |
| Suspended from school in past year ^a^ | School | Step 3 | Significant |
| *Note.* Covariates grouped according to key area associated with early school leaving.  ^a^ Covariate retained in final regression model. | | | |
